# Kullback–Leibler divergence and the Pareto–Exponential approximation

**DOI:** 10.1186/s40064-016-2253-y

**Published:** 2016-05-12

**Authors:** G. V. Weinberg

**Affiliations:** Defence Science and Technology Group, Edinburgh, Australia

## Abstract

Recent radar research interests in the Pareto distribution as a model for X-band maritime surveillance radar clutter returns have resulted in analysis of the asymptotic behaviour of this clutter model. In particular, it is of interest to understand when the Pareto distribution is well approximated by an Exponential distribution. The justification for this is that under the latter clutter model assumption, simpler radar detection schemes can be applied. An information theory approach is introduced to investigate the Pareto–Exponential approximation. By analysing the Kullback–Leibler divergence between the two distributions it is possible to not only assess when the approximation is valid, but to determine, for a given Pareto model, the optimal Exponential approximation.

## Background

The Pareto distribution has become important in maritime surveillance radar signal processing, since it has been validated as an intensity model for X-band clutter returns. Beginning with the work of Balleri et al. (2007), the Pareto model was fitted to data obtained by the Canadian IPIX radar, while situated at a test site located on Lake Ontario, in Grimsby Canada, with the radar located at a height of 20 m. This radar operated at a frequency of 8.9–9.4 GHz, with a pulse repetition frequency of 1000 Hz and compressed pulse length of 0.06 μs, resulting in a range resolution of 9 m. It operated in horizontal transmit and receive (HH), vertical transmit and receive (VV) as well as in the cross polarisation case of vertical transmit and horizontal receive (VH). It was reported that the Pareto fit improved on that of the Weibull, K and Log-Normal in all cases examined, especially in the HH polarised case.

A second validation of the Pareto model for sea clutter is given in Farshchian and Posner (2010), who describe and analyse sea clutter returns obtained during a United States’ Naval Research Laboratory (NRL)-led trial in 1994, located in Kuai, Hawaii. The radar operated at a frequency of 9.5–10 GHz with a pulse repetition frequency of 2000 Hz, 2.5 μs compressed pulse length and 0.375 m range resolution. It operated in both HH and VV polarisations; however, the radar used was not dual polarised and so these were collected separately. The radar was at a height of 23 m above sea level, so that the grazing angle was 0.22° and the radar range was 5.74 km for VV and 6.11 km for HH-polarisation. The data analysed in Farshchian and Posner (2010) focused on the up wind direction, which is generally the most spiky. The wind speed was roughly 9 m/s and the largest wave height was roughly 3 m, so that the sea state was approximately 4. The results of the trial was conclusive evidence that at a low grazing angle, the Pareto model outperformed the Weibull, Log-Normal and K-Distributions. Additionally, the model was compared to mixtures of Weibull and K, and shown to outperform Weibull mixtures, while having comparable performance to a K-mixture model. Given the latter is a three to four parameter model, the performance of the two parameter Pareto model was determined to be excellent.

A third validation for the Pareto model has been provided by Defence Science and Technology Group (DSTG) in Australia, based upon data from their Ingara radar. Ingara is an experimental fully polarimetric airborne multi-mode X-band imaging radar developed by DSTG (Stacy and Burgess 1994), which was deployed in a Raytheon Beech 1900C aircraft during a number of trials. A trial was conducted in 2004, in the Southern Ocean near Port Lincoln in South Australia (Stacy et al. 2005). The radar operated with a frequency of 10.1 GHz, with a pulse length of 20 μs, pulse repetition frequency of 300 Hz and LFM transmitted bandwidth of 200 MHz. This permitted a range resolution of 0.75 m. Ingara operated in a circular spotlight mode, surveying the same patch of ocean at all azimuth angles (0°–360°), and over the range of grazing angles 10°–45°. Sea states varied from 2 to 5, while wind speeds varied from 6.1 to 13.2 m/s. The data gathered in this trial was analysed in blocks composed of 1024 range compressed samples of roughly 920 pulses over 5° azimuth angle increments. The Pareto fit to the Ingara clutter has been reported initially in Weinberg (2011a), then further analysed in Rosenberg and Bocquet (2013). The inclusion of receiver thermal noise in the Ingara data, together with a Pareto clutter model, has also been reported in Rosenberg and Bocquet (2015). The conclusions from these investigations was that the Pareto distribution also fitted medium to high grazing angle clutter, obtained from an airborne surveillance radar.

These three independent studies confirmed the validity of the Pareto model for X-band maritime surveillance radar clutter, regardless of the radar platform and independent of the grazing angle. Consequently much effort has been invested in the development of non-coherent detection under a Pareto clutter model assumption (Weinberg 2013a, 2015).

The Pareto distribution also fits into the currently accepted framework for clutter models in the complex domain, since it arises as the intensity model of a compound Gaussian distribution with inverse Gamma texture (Weinberg 2011b). As a result of this, coherent radar detection schemes have been analysed extensively, based upon this clutter model assumption (Sangston et al. 2012; Shang and Song 2011; Weinberg 2013b, c).

Although the Pareto model has presented radar researchers with a simpler alternative to the Weibull and K-distributions, there is still merit in applying the original detection schemes designed for target detection in Gaussian clutter, or in Exponentially distributed intensity clutter, since in some cases X-band clutter is reasonably approximated by these processes. The validity of such an approximation has been analysed in Weinberg (2012), who investigated the Exponential approximation of a Pareto distribution with Stein’s Method. It was shown that relative to DSTG’s Ingara radar clutter, in the case of VV-polarisation, the Exponential approximation was valid. This coincided with Pareto fits to the data which resulted in large shape parameters. Stein’s Method was used to construct explicit bounds to quantify this observation.

The current paper is concerned with understanding the validity of the Pareto–Exponential approximation, through an analysis of the Kullback–Leibler divergence. This will be shown to not only provide a simpler estimate of the distributional difference, but also will indicate how an optimal Exponential distribution can be selected for any given Pareto model. Numerical comparisons are used to demonstrate the validity of the approach.

## Pareto and Exponential distributions

Before proceeding with the analysis of the Kullback–Leibler divergence, a brief overview of the relevant distributions is undertaken. A useful reference which contains details of these distributions is Beaumont (1980). A random variable *X* has a Pareto distribution with shape and scale parameters $\alpha >0$ and $\beta >0$ respectively if its probability density function is

1 $$\begin{aligned} f_{X}(t) = \frac{\alpha \beta ^\alpha }{ \left( t + \beta \right) ^{\alpha +1}} \end{aligned}$$

and its cumulative distribution function

2 $$\begin{aligned} F_{X}(t) = \mathbb {P}(X \le t) = 1 - \left( \frac{\beta }{t + \beta }\right) ^\alpha , \end{aligned}$$

where $t \ge 0$ and $\mathbb {P}$ denotes probability. Similarly, a random variable *Y* with shape parameter $\lambda >0$ has an Exponential distribution if its density is given by

3 $$\begin{aligned} f_Y(t) = \lambda e^{-\lambda t} \end{aligned}$$

and cumulative distribution function

4 $$\begin{aligned} F_Y(t) = \mathbb {P}(Y \le t) = 1 - e^{-\lambda t} \end{aligned}$$

also for $t\ge 0$. One of the fundamental differences between these distributions is the existence of moments. For the Pareto distribution, the existence of moments depends on the magnitude of its shape parameter, while for the Exponential distribution such moments always exist (Beaumont 1980). The problem of interest is to understand when (4) is a reasonable approximation for (2). Since, on the basis of empirical studies such as Weinberg (2011a), this will happen as the Pareto shape parameter increases, it will be assumed that $\alpha \gg 1$ throughout without loss of generality.

The Exponential distribution arises as a limit of the Pareto as the latter’s shape parameter increases. This can be seen through a reparameterisation of $\beta = \frac{\alpha (1 + o_\alpha (1))}{\lambda }$ where $o_\alpha (1) \rightarrow 0$ as $\alpha \rightarrow \infty$. Applying this to the complementary distribution function of *X* yields

5 $$\begin{aligned} \lim _{\alpha \rightarrow \infty } \mathbb {P}(X> t) &= \lim _{\alpha \rightarrow \infty }\left( 1 + \frac{t}{\beta }\right) ^{-\alpha } \nonumber \\ \nonumber \\ &= \lim _{\alpha \rightarrow \infty } \left( 1 + \frac{\lambda t}{\alpha (1 + o_\alpha (1))}\right) ^{-\alpha } \nonumber \\ &= e^{-\lambda t} = \mathbb {P}(Y>t), \end{aligned}$$

from which it can be concluded that the distribution function of *X* limits to that of *Y* as the Pareto shape parameter increases without bound.

The limit (5) can be quantified by establishing bounds on the distributional differences. Towards this aim, Stein’s Method (Barbour and Chen 2005) can be used to measure the rate of convergence of (2) to a limiting distribution of the form (4). This method starts with a differential equation characterising the Exponential distribution, and bounds on this are then used to measure the rate of convergence. In particular, it is shown in Weinberg (2012) that the two distributions above satisfy the inequality

6 $$\begin{aligned} -(1-e^{-\lambda }) \frac{1}{\alpha -1} \le F_X(t) - F_Y(t) \le \frac{3}{\alpha }, \end{aligned}$$

which shows that the rate of convergence is controlled by the Pareto shape parameter. It is clear that as $\alpha$ increases, the bounds in (6) decrease to zero, implying the Exponential approximation to the Pareto model is valid for large shape parameters.

The problem with the Stein approach is that the bounds do not suggest a suitable way in which, for a given Pareto model, an appropriate approximating Exponential distribution can be specified. This can be rectified with an application of the Kullback–Leibler divergence as an alternative to analysing distributional approximations.

## Information theory

Information theory is concerned with the study of entropy as a measure of uncertainty, and was introduced into the engineering community by Shannon (1948), and has had a profound effect on the understanding and optimisation of data networks (Arndt 2004). In particular, the Kullback–Leibler divergence, introduced in Kullback and Leibler (1951), has found application in signal processing analysis and statistical model fitting (Hulle 2005; Seghouane 2006; Youssef et al. 2016; Wenling and Yingmin 2016).

The Kullback–Leibler divergence is a measure of the information lost when one distribution is approximated by another. Hence, for two random variables *X* and *Y* with densities $f_X$ and $f_Y$, the information lost when *Y* is used to approximate *X* is defined to be

7 $$\begin{aligned} D_{KL}(X || Y) := \int _{0}^\infty f_{X}(t) \log \left( \frac{f_{X}(t)}{f_Y(t)}\right) dt, \end{aligned}$$

where it has been assumed that these two random variables have support the nonnegative real line. It can be shown that (7) is the difference between the cross entropy of *X* and *Y*, and the entropy of *X* (Arndt 2004). Since (7) measures the information lost in the approximation of *X* by *Y*, it can be used to assess the convergence of these distributions.

It can be shown that $D_{KL}(X ||Y) \ge 0$, a result known as Gibb’s Inequality, which follows from Jensen’s Inequality (Arndt 2004). It is clear that if the two random variables *X* and *Y* coincide then $D_{KL}(X ||Y) =0$. The converse of this can also be demonstrated to be true. However, it is clear from (7) that the Kullback–Leibler divergence is not symmetric, nor satisfies a triangle inequality. Consequently it is not a metric but is a pseudo-metric. Its value in assessing convergence in distribution follows from the Pinsker–Csiszár Inequality (Pinsker 1964; Csiszár 1967; Kullback 1967). Suppose for the two random variables *X* and *Y* their distribution functions are $F_X(t)$ and $F_Y(t)$ respectively, with support the nonnegative real line. Then this inequality states that

8 $$\begin{aligned} \Vert F_X - F_Y\Vert _{\infty } = \sup _{t\ge 0} \left| F_X(t) - F_Y(t)\right| \le \sqrt{ 2 D_{KL}(X ||Y)}, \end{aligned}$$

where the norm on the left hand side of (8) is the supremum norm over the domain of the distribution functions. Clearly if the Kullback–Leibler divergence is close to zero, the supremum norm inherits this and thus implies the random variables *X* and *Y* are close in distribution.

Also based upon (8), if a sequence of random variables $X_{n}$ is such that $\lim\nolimits_{n\rightarrow \infty } D_{KL}(X_{n} || Y) = 0$, for some random variable *Y*, then the limiting distribution of $X_n$ and *Y* coincide, which can be justified with an application of Lebesgue’s Dominated Convergence Theorem.

These results justify using the Kullback–Leibler divergence to measure distributional approximations. It is worth noting that although the triangle inequality is not achievable with this divergence, it is possible to construct a measure which is symmetric. This can be produced by defining the distance

9 $$\begin{aligned} {\widetilde{D}}_{KL}(X || Y) = D_{KL}(X ||Y) + D_{KL}(Y ||X), \end{aligned}$$

which has been utilised in Seghouane (2006). However, as will be shown in the next section, it is sufficient to apply (7) to the problem under investigation.

## Kullback–Leibler divergence

This section calculates the Kullback–Leibler divergence (7) for the two statistical models of interest. With an application of (1) and (3), observe that

10 $$\begin{aligned} \frac{f_X(t)}{f_Y(t)} = \frac{\alpha \beta ^\alpha }{\lambda } e^{\lambda t} (t+\beta )^{-\alpha -1}, \end{aligned}$$

from which it follows by applying logarithms to (10) and substituting the result into (7) that

11 $$\begin{aligned} D_{KL}(X || Y) = \log \left( \frac{\alpha \beta ^\alpha }{\lambda }\right) + \lambda \mathbb {E}(X) - (\alpha +1) \mathbb {E}(\log (X+\beta )), \end{aligned}$$

where $\mathbb {E}$ is the statistical mean with respect to the distribution of *X*, and the fact that the density of *X* integrates to unity has been applied.

The mean of *X* can be shown to be

12 $$\begin{aligned} \mathbb {E}(X) = \frac{\beta }{\alpha -1}, \end{aligned}$$

with the proviso that $\alpha > 1$, while the mean of $\log (X+\beta )$ is given by

13 $$\begin{aligned} \mathbb {E}(\log (X+\beta )) = \int _0^\infty \log (t+\beta ) \frac{\alpha \beta ^\alpha }{(t+\beta )^{\alpha +1}}dt. \end{aligned}$$

By applying a transformation $u = \log (t+\beta )$, followed by integration by parts, it can be shown that (13) reduces to

14 $$\begin{aligned} \mathbb {E}(\log (X+\beta )) = \log (\beta ) + \frac{1}{\alpha }. \end{aligned}$$

An application of (12) and (14)–(11) demonstrates that the Kullback–Leibler divergence reduces to

15 $$\begin{aligned} D_{KL}(X||Y) = \log \left( \frac{\alpha }{\lambda \beta }\right) + \frac{\lambda \beta }{\alpha -1} - \left( \frac{\alpha +1}{\alpha }\right) . \end{aligned}$$

**Fig. 1:**
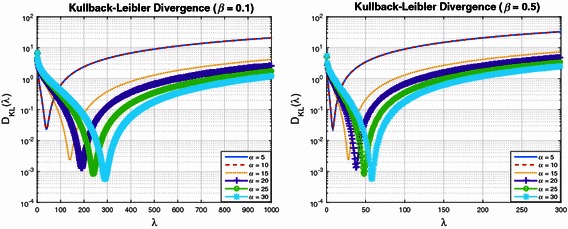
Kullback–Leibler divergence as a function of the Exponential distribution shape parameter $\lambda$

**Fig. 2:**
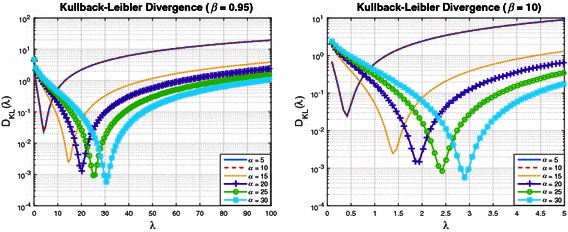
Further examples of Kullback–Leibler divergence as a function of $\lambda$

Figures 1 and 2 plot the Kullback–Leibler divergence (15) as a function of $\lambda$, for a series of Pareto shape and scale parameters. Each figure shows curves for a specified $\beta$, with $\alpha \in \{5, 10, 15, 20, 25, 30\}$. Figure 1 is for the case where $\beta = 0.1$ (left subplot) and $\beta = 0.5$ (right subplot). Figure 2 is for $\beta = 0.95$ (left subplot) and $\beta = 10$ (right subplot). These figures show a common structure to the Kullback–Leibler divergence. In particular, for each $\alpha$ and $\beta$ there exists a $\lambda$ which minimises (15). It is also interesting to observe the effect $\beta$ has on the Kullback–Leibler divergence. For a target upper bound of approximately $10^{-3}$ on the Kullback–Leibler divergence, it is clear from Fig. 1 (left subplot) that for $\alpha \approx 30$, one must select $\lambda \approx 300$. For the case of $\beta = 0.5$, Fig. 1 (right subplot) suggests that $\alpha \approx 30$ and $\lambda \approx 50$. For the case of $\beta = 0.95$, Fig. 2 (left subplot) suggests that $\alpha \approx 30$ and $\lambda \approx 30$. Finally, as shown in the right subplot of Fig. 2, for $\beta = 10$ we require $\alpha \approx 30$ and $\lambda \approx 3$.

To understand this mathematically, differentiating (15) with respect to $\lambda$ yields

16 $$\begin{aligned} \frac{\partial D_{KL}(X||Y)}{\partial \lambda } = -\frac{1}{\lambda } + \frac{\beta }{\alpha -1}, \end{aligned}$$

which is zero when $\lambda = \frac{\alpha -1}{\beta }$, where it is necessary to assume $\alpha > 1$. Applying a second differentiation to (16) shows that this is a point where a minimum occurs. This explains the phenomenon observed in these plots.

**Fig. 3:**
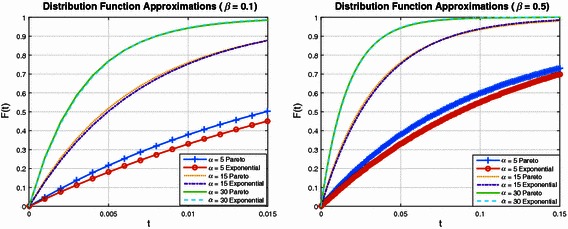
Comparison of distributions where the Exponential has shape parameter selected so that it is the optimal fit for each Pareto case

**Fig. 4:**
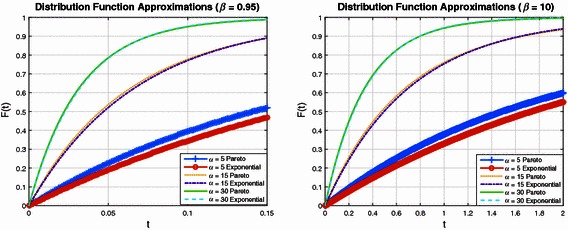
Further examples of distributional approximations

In order to investigate these results further, Figs. 3 and 4 plot a series of Pareto distributions, together with the optimal Exponential approximation. Here optimal is used in the sense that the Kullback–Leibler divergence is minimised with an appropriate selection of Exponential distribution shape parameter. Figure 3 (left subplot) is for the case where the Pareto scale parameter is $\beta = 0.1$, with shape parameter varying from 5, 15 to 30. It can be observed that as the Pareto shape parameter increases, the optimal Exponential distribution is a better fit. This is consistent with the results illustrated in Fig. 1. Figure 3 (right subplot) is for the case where $\beta = 0.5$, Fig. 4 (left subplot) corresponds to $\beta = 0.95$ and Fig. 4 (right subplot) is for $\beta = 10$. Observe in all figures that for $\alpha = 5$, the approximation is poor, while for $\alpha = 15$ the approximation has improved significantly. When $\alpha = 30$ it is very difficult to see a difference between the two distributions.

Returning to the analysis of the minimum achievable divergence, by applying $\lambda = \frac{\alpha -1}{\beta }$ to (15), it can be shown that the minimum divergence is

17 $$\begin{aligned} D_{KL}(X||Y)_{\min } = \log \left( 1 + \frac{1}{\alpha -1}\right) - \frac{1}{\alpha }. \end{aligned}$$

Since for any $x>0$ we have the bound $\log (1+x) \le x$, an application of this to (17) yields the upper bound

18 $$\begin{aligned} D_{KL}(X||Y)_{\min } \le \frac{1}{\alpha (\alpha -1)}. \end{aligned}$$

An application of (18)–(8) results in

19 $$\begin{aligned} \Vert F_X - F_Y\Vert _{\infty } \le \sqrt{\frac{2}{\alpha (\alpha -1)}}. \end{aligned}$$

One can compare the upper bound provided by (19) to that obtained via Stein’s Method, given by the upper bound $\frac{3}{\alpha }$ in (6). With an application of some simple analysis one can show that the upper bound based upon (19) improves on that from (6) whenever $\alpha ^2 - \frac{9}{7}\alpha > 0$. This occurs when $\alpha > \frac{9}{7}$, and since in most cases $\alpha > 2$, as shown in Weinberg (2011a), it follows that the upper bound attained by the Kullback–Leibler divergence is smaller than that obtained with Stein’s Method.

**Fig. 5:**
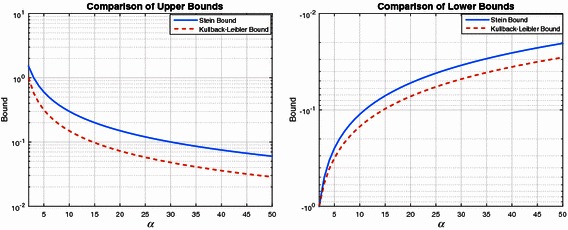
Comparison of upper and lower bounds for Pareto–Exponential approximation

To illustrate the differences between the upper bounds, Fig. 5 (left subplot) plots the two upper bounds as a function of $\alpha$. It can be observed that the upper bound (19) is better than that based upon (6).

Using a similar analysis it can be shown that the Stein lower bound, namely $-\frac{1}{\alpha -1}$, tends to be closer to zero than that obtained by the Kullback–Leibler divergence, as illustrated in the right subplot of Fig. 5.

## Conclusions

The Kullback–Leibler divergence was used to assess the discrepancy between the Pareto and Exponential distributions, in order to better understand the validity of the Exponential approximation of the Pareto model. It was shown that for any given Pareto model an optimal Exponential approximation exists. This approximation was shown to improve as the Pareto shape parameter increased, for any fixed Pareto scale parameter. This means that in cases where in X-band maritime surveillance radar the Pareto shape parameter exceeds 30, it is acceptable to apply detection schemes based upon an Exponential clutter model assumption.

## References

[CR1] Arndt C (2004). Information measures: information and its description in science and engineering.

[CR2] Balleri A, Nehorai A, Wang J (2007). Maximum likelihood estimation for compound-Gaussian clutter with inverse Gamma texture. IEEE Trans Aerosp Electron Syst.

[CR3] Barbour AD, Chen LHY (2005). An introduction to Stein’s method.

[CR4] Beaumont GP (1980). Intermediate mathematical statistics.

[CR5] Csiszár I (1967). Information-type measures of difference of probability distributions and indirect observations. Stud Sci Math Hung.

[CR6] Farshchian M, Posner FL (2010) The Pareto distribution for low grazing angle and high resolution X-band sea clutter. In: IEEE Radar conference, pp 789–793

[CR7] Van Hulle MM (2005). Mixture density modeling, Kullback–Leibler divergence, and differential log-likelihood. Signal Process.

[CR8] Kullback S (1967). Lower bound for discrimination information in terms of variation. IEEE Trans Inf Theory.

[CR9] Kullback S, Leibler RA (1951). On information and sufficiency. Ann Math Stat.

[CR10] Pinsker MS (1964) Information and information stability of random variables and processes (trans: Feinstein A). USSR series Problemy Peredaci Informacii Moscow 1960, vol 7. Holden-Day, San Francisco

[CR11] Rosenberg L, Bocquet S (2015). Application of the Pareto plus noise distribution to medium grazing angle sea-clutter. IEEE Sel Top Appl Earth Obs Remote Sens.

[CR12] Rosenberg L, Bocquet S (2013) The Pareto distribution for high grazing angle sea-clutter. In: Proceedings of international geoscience and remote sensing symposium, pp 4201–4212

[CR13] Sangston KJ, Gini F, Greco MS (2012). Coherent radar target detection in heavy-tailed compound Gaussian clutter. IEEE Trans Aerosp Electron Syst.

[CR14] Seghouane A-K (2006). Multivariate regression model selection from small samples using Kullback’s symmetric divergence. Signal Process.

[CR15] Shang X, Song H (2011). Radar detection based on compound-Gaussian model with inverse gamma texture. IET Radar Sonar Navig.

[CR16] Shannon CE (1948). A mathematical theory of communication. Bell Syst Tech J.

[CR17] Stacy NJS, Burgess MP (1994) Ingara: the Australian airborne imaging radar system. In: Proceedings of the international geoscience and remote sensing symposium, pp 2240–2242

[CR18] Stacy N, Crisp D, Goh A, Badger D, Preiss M (2005) Polarimetric analysis of fine resolution X-band sea clutter data. In: Proceedings of the international geoscience and remote sensing symposium, pp 2787–2790

[CR19] Weinberg GV (2011). Assessing Pareto fit to high resolution high grazing angle sea clutter. IET Electron Lett.

[CR20] Weinberg GV (2011). Coherent multilook radar detection for targets in Pareto distributed clutter. IET Electron Lett.

[CR21] Weinberg GV (2012). Validity of whitening-matched filter approximation to the Pareto coherent detector. IET Signal Process.

[CR22] Weinberg GV (2013). Constant false alarm rate detectors for Pareto cutter models. IET Radar Sonar Navig.

[CR23] Weinberg GV (2013). Assessing detector performance, with application to Pareto coherent multilook radar detection. IET Radar Sonar Navig.

[CR24] Weinberg GV (2013). Coherent CFAR detection in compound Gaussian clutter with inverse gamma texture. EURASIP Adv Signal Process.

[CR25] Weinberg GV (2015). Examination of classical detection schemes for targets in Pareto distributed clutter: do classical CFAR detectors exist, as in the Gaussian case?. Multidimens Syst Signal Process.

[CR26] Wenling L, Yingmin J (2016). Kullback-Leibler divergence for interacting multiple model estimation with random matrices. IET Signal Process.

[CR27] Youssef A, Delpha C, Diallo D (2016). An optimal fault detection threshold for early detection using Kullback–Leibler divergence for unknown distribution data. Signal Process.

